# Serial Optical Coherence Tomography Findings After Lesion-Preparation Incorporating the ShockFast™ Intravascular Lithotripsy: A Prospective Single-Center Imaging Sub-Study

**DOI:** 10.3390/life16101659

**Published:** 2026-10-02

**Authors:** Giacomo Maria Cioffi, Ioannis Skalidis, Julius Jonas Jelisejevas, Yannick Faucherre, Peter Wenaweser, Pascal Meier, Mario Togni, Stéphane Cook

**Affiliations:** Department of Cardiology, University and Hospital Fribourg,1708 Fribourg, Switzerland

**Keywords:** intravascular lithotripsy, optical coherence tomography, coronary calcification, ShockFast™, calcium fracture, stent expansion, percutaneous coronary intervention

## Abstract

**Background:** Intravascular lithotripsy (IVL) is an established calcium modification strategy for severely calcified coronary lesions. The Shunmei ShockFast™ IVL system is a novel device that appeared safe and feasible in an initial clinical experience. However, its calcium fracture typology, depth distribution, and quantitative stent optimization characteristics have not been characterized by serial optical coherence tomography (OCT). **Objectives:** To provide the first dedicated serial OCT sub-study of calcium modification patterns and stent optimization metrics achieved with the ShockFast™ IVL system in a cohort of patients with severely calcified de novo coronary lesions. **Methods:** This is a prospective, single-center OCT imaging sub-study nested within the Cardio-FR registry (NCT04185285). We included 14 patients with OCT assessment performed at three mandatory time points—baseline (before any device delivery), post-IVL, and post-stenting–who underwent PCI with the ShockFast™ IVL system between June 2025 and April 2026. The primary endpoint was OCT-confirmed calcium fracture rate following IVL treatment. Secondary endpoints included calcium fractures ≥2 and type, post-lesion-preparation lumen gain, minimum stent area (MSA), stent expansion (SE), malapposition, and edge dissection rates. Exploratory correlative analyses between baseline calcium morphology and post-IVL stent optimization outcomes were performed. **Results:** Median baseline OCT MLA was 2.22 mm^2^ (IQR 1.4–3.61). Calcium fractures were identified in 10 of 14 lesions (71%, 95% CI 45–88%) following a lesion-preparation strategy incorporating ShockFast™ IVL, with ≥2 fractures in 43%. Fractures were classified as partial-thickness (non-transmural) in 30% and full-thickness (transmural) in 70% of cases. Median post-lesion-preparation lumen gain was 0.53 mm^2^ (IQR 0.3–0.84). Median final MSA was 4.77 mm^2^ (IQR 3.59–5.34) and median SE was 89.5% (IQR 85–94), with SE >80% achieved in 100% of lesions. No major malapposition or medial edge dissections were recorded. **Conclusions:** Applied as the central calcium-modification component of a comprehensive lesion-preparation strategy, the ShockFast™ IVL system was associated with OCT-confirmed calcium fractures in the majority of the lesions, with favorable stent expansion and optimization metrics in the absence of major malapposition or edge dissections. These findings provide the first dedicated serial OCT sub-study of calcium modification mediated by a lesion-preparation strategy incorporating ShockFast™ IVL, consistent with feasibility as a novel IVL platform suitable for further investigation in adequately powered comparative studies.

## 1. Introduction

Severe coronary artery calcification represents one of the most challenging substrates encountered in percutaneous coronary intervention (PCI), carrying an elevated risk of procedural failure, stent underexpansion, and adverse long-term outcomes including in-stent restenosis and stent thrombosis [1,2,3,4,5]. Calcium deposits within the coronary vessel wall–whether superficial, deep, or organized as calcified nodules–impair vessel compliance, resist balloon expansion, and compromise the uniform apposition and expansion of drug-eluting stents (DES) [5,6]. These limitations have driven the development of dedicated calcium modification strategies to enable adequate lesion preparation and optimize stent deployment in this complex patient subset [7].

Intravascular lithotripsy (IVL) has emerged as a transformative calcium modification technology, delivering pulsatile acoustic pressure waves via a balloon catheter to selectively fracture calcified plaque while largely preserving surrounding soft tissue [8]. The Shockwave™ C2 IVL system (Shockwave Medical, Santa Clara, CA, USA) has accumulated robust evidence across dedicated trials and real-world registries, consistently demonstrating high procedural success, favorable safety profiles, and–critically–OCT-confirmed calcium fracture rates of 79–82% with multiple fractures in 52–67% of treated lesions [9,10,11,12,13]. These OCT sub-studies have been pivotal in establishing that acoustic-mediated calcium fractures are the structural basis for the improved vessel compliance and stent optimization observed following IVL treatment [14,15].

Optical coherence tomography (OCT) is the gold-standard intracoronary imaging modality for the characterization of coronary calcification, providing near-histological resolution to identify and quantify calcium arc, thickness, length, and depth [16,17]. OCT-derived calcium scoring systems have been validated to predict stent underexpansion risk prior to intervention [18,19], and serial OCT assessment across the procedural timeline allows direct visualization of calcium fractures, quantification of post-lesion-preparation lumen gain, and detection of stent optimization endpoints including expansion index, malapposition, and edge dissections [14,15]. The integration of systematic OCT guidance with IVL is therefore uniquely suited to provide a comprehensive mechanistic and outcome-oriented assessment of novel IVL platforms.

The Shunmei ShockFast™ IVL system (Shunmei Medical, Shenzhen, China), which received CE marking in May 2025, represents a novel IVL platform with a distinct technical design incorporating two lithotripsy emitters per catheter. In our recent first-in-center experience (16 patients, OCT performed in 8), we reported 100% procedural success and the presence of calcium fractures in the OCT-assessed subpopulation, without serial pullback analysis, fracture typology or depth distribution, quantitative stent optimization metrics, or morphology–outcome analyses [20]. The present study comprises 14 patients with complete three-time-point OCT, of whom the 8 previously OCT-assessed patients are included together with 6 newly enrolled OCT-guided patients. It is, to our knowledge, the first dedicated serial OCT sub-study of the ShockFast™ system (defined by the three-time-point protocol and quantitative pullback-to-pullback analysis) providing a systematic assessment of calcium modification and stent optimization following a lesion-preparation strategy incorporating ShockFast™ IVL in severely calcified de novo coronary lesions.

## 2. Methods

### 2.1. Study Design and Population

This is a prospective, single-center, imaging-focused observational sub-study nested within the ongoing Cardio-FR registry (NCT04185285), conducted at the Department of Cardiology, University and Hospital of Fribourg, Switzerland. The registry was initiated in January 2015, is approved by the local ethics committee of Swissethics (003-REP-CER-FR, approved 1 May 2015), and is conducted in accordance with the Declaration of Helsinki. All patients provided written informed consent prior to enrollment. The present sub-study prospectively included consecutive patients undergoing PCI for severely calcified de-novo lesions with the ShockFast™ IVL system between June 2025 and April 2026 in whom OCT was performed at three pre-specified mandatory time points. Eight patients in the present sub-study were also included in our previously reported first-in-center experience with the ShockFast™ system [20]; the remaining 6 patients were enrolled subsequently. The present sub-study includes only patients with complete three-time-point OCT acquisition and presents a dedicated imaging analysis not reported in the prior publication. Eight patients in the present cohort were included in our prior first-in-center report [20]; the present sub-study adds six newly enrolled OCT-guided patients and, for all 14, a serial three-time-point OCT protocol and quantitative analyses not previously reported.

### 2.2. Eligibility Criteria

Angiographic inclusion criteria were: at least one de novo coronary stenosis >50% of diameter, lesion length ≤50 mm, distal reference vessel diameter 2.0–4.0 mm by visual estimation, and angiographic evidence of severe coronary calcification per the modified ACC/AHA classification [21,22,23]. OCT inclusion criteria required a revised OCT calcium score ≥2 points [19] or an original OCT calcium score >3 points [18]. For the purposes of this sub-study, only patients with complete OCT assessment at all three mandatory pullback time points were included in the primary imaging analysis. Exclusion criteria were: severe acute or chronic renal impairment, cardiogenic shock, severe chronic obstructive pulmonary disease, and contrast allergy.

### 2.3. Study Device

The ShockFast™ coronary IVL system (Shunmei Medical, Shenzhen, China) received CE marking on 18 May 2025. It is a 0.014-inch guidewire-compatible, contrast fluid-filled balloon angioplasty catheter incorporating two lithotripsy emitters within a 12 mm or 15 mm balloon segment, available in diameters of 2.5, 2.75, 3.0, 3.25, 3.5, and 4.0 mm. Each catheter is intended for single use and delivers up to 120 total pulses in cycles of 10, at a balloon treatment pressure of 4 atm, nominal pressure of 6 atm, and rated burst pressure of 10 atm.

### 2.4. Interventional Procedure and OCT Protocol

The PCI procedure has been described in detail previously [20]. Briefly, the IVL catheter was sized 1:1 to the distal reference vessel diameter. Predilatation between baseline OCT (Pullback 1) and IVL delivery was performed at the operator’s discretion, primarily to facilitate ShockFast™ IVL balloon delivery in severely calcified lesions; it was not part of a routine pre-specified algorithm. IVL was repeated until full balloon expansion was achieved, or all pulses were depleted. Additional non-compliant balloon (NCB) post-dilatation followed IVL treatment and preceded stent implantation in all cases. Multiple contemporary drug-eluting stent platforms were used across the cohort, reflecting our institution’s daily-rotation vendor inventory; ultra-thin-strut platforms (e.g., Orsiro, Biotronik) were avoided due to severely calcified coronary lesions. Dual antiplatelet therapy was prescribed per current ESC guidelines [24].

OCT imaging was performed using the Ultreon™ 2.0 OPTIS™ system with the OPTIS™ Integrated Catheter (Abbott Vascular, Santa Clara, CA, USA). OCT pullbacks were performed in the following procedural sequence: Pullback 1 (baseline) was acquired before any device delivery, including before any predilatation; Pullback 2 was acquired immediately following completion of IVL treatment, prior to any additional balloon dilatation or stent implantation; Pullback 3 was acquired following final DES deployment and non-compliant balloon post-dilatation. Predilatation, when performed, occurred between Pullbacks 1 and 2. Additional pullbacks between procedural steps were encouraged but were ultimately at operator discretion. All OCT images were analyzed offline using the Ultreon™ 2.0 OPTIS™ Software (Abbott Vascular).

### 2.5. OCT Image Analysis

OCT image analysis was performed offline by the lead investigator (G.M.C.). Images were recalibrated when needed using the dedicated software adjustment tool. The following quantitative measurements were collected at baseline (Pullback 1): minimum lumen area (MLA, mm^2^), minimum lumen diameter (MLD, mm), proximal and distal reference mean lumen diameter (mm), proximal and distal external elastic lamina (EEL) mean diameter (mm) measured at 5 mm from the lesion edges, and lesion length (mm). OCT lesion length was defined as the longitudinal segment between the proximal and distal OCT reference frames in which the lumen area was ≥10% smaller than the average of the two reference lumen areas. OCT calcium length was defined as the longitudinal extent of any OCT-identified calcium (signal-poor regions with sharp borders) of any thickness or arc, irrespective of lumen dimensions. The maximum calcification site was defined as the OCT cross-section showing the largest calcium arc within the lesion; calcium arc (°) and calcium thickness (mm) were measured at this cross-section.

Post-lesion-preparation OCT (Pullback 2) measurements included: post-lesion-preparation lumen gain (mm^2^), defined as (Pullback 2 MLA–Pullback 1 MLA); presence and number of calcium fractures—defined as a discontinuity/break in previously continuous calcium with separation of edges—and fracture depth classification. Calcium fracture depth was classified per lesion at the maximum calcium site using the deepest fracture observed when multiple fractures were present, using a streamlined, three mutually exclusive categories’ method: (1) partial-thickness (non-transmural), defined as a fracture clearly not reaching the abluminal calcium border; (2) full-thickness (transmural) fracture, defined as a fracture extending from the luminal to the abluminal border of the deposit; and (3) indeterminate, defined in cases where any fracture or the abluminal border were not visualized due to OCT signal penetration limitation. Compared with the more granular per-cross-section assessment used by core laboratories (measurement of calcium fracture width, depth, arc and length at each OCT cross section), this streamlined per-lesion classification was adopted to maintain methodological feasibility for an institutional imaging sub-study while preserving the principal mechanistic distinction between non-transmural and transmural fracture.

Post-stenting and optimization (Pullback 3) measurements included: minimum stent area (MSA, mm^2^), stent expansion (SE, %) defined as the ratio of MSA to average proximal and distal reference lumen area, and acute lumen gain (mm^2^) defined as the increase in lumen area between baseline MLA and the final lumen area at the MSA site [17].

Qualitative characteristics collected included: presence and type of calcified nodule (eruptive vs. non-eruptive), original OCT calcium score [18], revised OCT calcium score [19], and presence of proximal and distal medial edge dissections. Malapposition was assessed per frame across the entire stented segment. Major malapposition was defined as a strut-to-wall distance >400 µm extending over a longitudinal length >1 mm, as per the European Association of Percutaneous Cardiovascular Interventions expert consensus on intracoronary imaging [17]. Minor malapposition was defined as any strut-to-wall distance exceeding the expected DES strut and polymer thickness but not meeting the major criterion. Side-branch-related floating struts and struts overlying calcium fracture gaps—in which strut-to-wall separation reflects the underlying calcium discontinuity rather than true incomplete apposition were excluded from the major-malapposition determination [17].

The presence and depth classification of calcium fractures was independently re-reviewed by a second investigator (J.J.J.) blinded to the initial reading; interobserver agreement was κ = 1.00 (100% agreement) on fracture presence in all 14 lesions and on fracture-depth classification in all 10 fracture-positive lesions. As no disagreement was observed, a formal kappa statistic was not reported. The analysts were not blinded to procedural outcomes, as both were involved in the index procedures. No independent core-laboratory adjudication was performed.

### 2.6. Endpoints

The primary endpoint was the presence of OCT-confirmed calcium fractures at the maximum calcium site following the complete lesion-preparation sequence culminating in ShockFast™ IVL (Pullback 2). Because OCT was not acquired between predilatation or atherectomy and IVL, this endpoint reflects the cumulative effect of lesion preparation rather than the isolated contribution of ShockFast™ IVL. Secondary endpoints included: proportion of lesions with ≥2 fractures, fracture depth distribution, post-lesion-preparation lumen gain (Pullback 2–Pullback 1), final MSA, SE (%), proportion of lesions achieving SE >80%, acute lumen gain, rate of major malapposition, and rate of medial edge dissections. Exploratory correlative analyses were pre-specified to evaluate relationships between baseline OCT calcium morphology parameters (calcium arc, thickness, length, OCT calcium score) and post-IVL outcomes (fracture presence, ≥2 fracture presence, final SE, MSA).

### 2.7. Statistical Analysis

Categorical variables are reported as absolute numbers and percentages. Continuous variables are reported as means ± standard deviation (SD) for normally distributed variables, or as medians with interquartile range bounds (Q1–Q3) for non-normally distributed variables, based on normality assessment using the Shapiro-Wilk test. Proportions are reported with 95% confidence intervals calculated using the Wilson score method. Spearman’s rank correlation coefficient (r_s_) was used to evaluate associations between continuous OCT variables. Given the limited sample size and the number of pairwise associations examined, these correlative analyses were pre-specified as exploratory and hypothesis-generating only; no correction for multiple comparisons was applied, and reported *p*-values are exact and descriptive, not confirmatory. No formal sample size calculation was performed, as this is an exploratory imaging sub-study; the cohort comprises all consecutive patients with complete three-time-point OCT during the enrolment window. Analyses were complete-case; no imputation was performed. Interobserver agreement for calcium fracture presence and depth was assessed using Cohen’s κ. All analyses were performed using STATA version 17 (StataCorp LLC, College Station, TX, USA).

## 3. Results

### 3.1. Study Population and Procedural Overview

Among 25 consecutive patients undergoing PCI with the ShockFast™ system at our institution between June 2025 and April 2026, 14 (56%) had OCT acquired at all three mandatory pullback time points and constitute the present imaging sub-study cohort. The remaining 11 patients had either no OCT performed or had incomplete OCT acquisition (most often a missing pre-IVL or post-IVL pullback) or non-diagnostic image quality precluding reliable quantitative analysis (Figure 1). These exclusions reflected operator-dependent variability in OCT use rather than patient-related factors. A formal comparison of included versus excluded patients was precluded by the absence of protocolized data collection in patients who did not undergo complete OCT; residual selection bias therefore cannot be excluded. Baseline clinical and demographic characteristics of the sub-study cohort are summarized in Table 1. The median age was 74 years, 79% were male, 29% had diabetes mellitus, and 93% presented with a chronic coronary syndrome. Angiographic and procedural characteristics are presented in Table 2 and Table 3. The main treated vessel was the RCA (71%, *n* = 10), and 93% (13/14) of lesions were classified as AHA/ACC type C with 1 lesion (7%) classified as type B2. ShockFast™ delivery was successful in all 14 cases. No failures to cross the target lesion with the IVL catheter, no instances requiring guide-extension support specifically for ShockFast™ delivery, no IVL balloon ruptures, and no incomplete IVL balloon expansions were observed. Procedural success was 100% in all OCT sub-study patients, with no intraprocedural complications, abrupt vessel closure, or in-hospital MACE.

### 3.2. Baseline OCT Calcium Characterization

OCT confirmed severe calcification in all lesions with a median original OCT calcium score of 4 and a median revised OCT calcium score of 3 (Table 4). Median calcium arc at the site of maximum calcification was 272° (IQR 200–308°), with a calcium arc exceeding 270° in 9 of 14 lesions (64%); median calcium thickness was 1.6 mm and median calcium length was 26.2 mm. Calcified nodules were identified in 8 (57%) patients, of which 5 (36%) were of the eruptive type. Median baseline MLA was 2.22 mm^2^ (IQR 1.4–3.61) and median MLD was 1.58 mm. Complete baseline OCT quantitative data are presented in Table 4.

### 3.3. Post-IVL OCT: Calcium Fracture Assessment

Following the lesion-preparation sequence culminating in ShockFast™ IVL, OCT confirmed calcium fractures in 10 of 14 lesions (71%, 95% CI 45–88%) at the site of maximum calcification, with presence of ≥2 fractures in 6 (43%) patients. Regarding fracture depth among the 10 fracture-positive lesions, 3 (30%) were classified as partial-thickness (non-transmural), and 7 (70%) as full-thickness (transmural), as depicted in Figure 2. A median post-lesion-preparation lumen gain of 0.53 mm^2^ (IQR 0.3–0.84) was observed during the pullback immediately after IVL use and prior to any additional balloon dilatation or stent implantation. In the 2 lesions that did not undergo predilatation, post-lesion-preparation lumen gain reflected the isolated effect of IVL and was 2.04 and 2.2 mm^2^. Additional data are presented in Table 5.

### 3.4. Post-Stenting OCT: Stent Optimization Metrics

Following DES implantation and optimization, final OCT assessment demonstrated a median MSA of 4.77 mm^2^ (IQR 3.59–5.34) and a median SE of 89.5% (IQR 85–94). SE >80% was achieved in all patients. Median acute lumen gain was 2.52 mm^2^ (IQR 1.49–3.57). No major malapposition (strut-to-wall distance >400 µm over >1 mm) was detected in any case. Minor malapposition was observed in 6 (43%) cases. No proximal or distal medial edge dissections were recorded. Complete stent optimization data are presented in Table 6.

Final stent optimization metrics were broadly comparable between fracture-positive (*n* = 10) and fracture-negative (*n* = 4) lesions: median MSA was 4.96 mm^2^ vs 3.69 mm^2^, and median stent expansion was 89.5% vs 91.5%, respectively. Consistent with the published Shockwave™ OCT experience [14,15], the presence of OCT-confirmed fractures was not associated with a measurable difference in final MSA in the present cohort–a finding that should be interpreted cautiously given the very small subgroup sizes. Among the 8 lesions with calcified nodules, median MSA was 4.31 mm^2^ and stent expansion 91.5%; among the 6 lesions without calcified nodules, median MSA was 4.83 mm^2^ and stent expansion 89.5%.

### 3.5. Exploratory Correlative Analyses

Spearman correlative analyses between baseline OCT calcium morphology parameters and post-IVL stent optimization outcomes are presented in Table 7. None of the baseline calcium morphology variables—including calcium arc, calcium thickness, calcium length, or OCT calcium scores (original or revised)—showed a meaningful correlation with final SE or fracture presence. A positive non-significant trend was observed between calcium thickness and final MSA (r_s_ = 0.51, *p* = 0.06), a finding that did not reach statistical significance in the present small cohort.

In this exploratory analysis the strongest observed association was a positive correlation between post-lesion-preparation lumen gain (Pullback 2–Pullback 1) and final MSA (r_s_ = 0.85, *p* = 0.0002, uncorrected and descriptive). This finding is hypothesis-generating only: in a 14-patient cohort no multivariable adjustment for vessel size, baseline MLA, reference lumen area, stent diameter, stent length, post-dilatation strategy, or atherectomy use was feasible, and the correlation may partly reflect shared dependence on vessel size and overall lesion expandability rather than a causal relationship between IVL-mediated lumen enlargement and final stent optimization. Confirmation in larger, adequately powered datasets with appropriate adjustment is required before this observation can inform clinical decision-making.

## 4. Discussion

In this first dedicated serial OCT sub-study of the Shunmei ShockFast™ IVL system, we provide a mechanistic characterization of calcium modification patterns and stent optimization metrics in 14 patients who underwent PCI for severely calcified de novo coronary lesions at a single center. Our principal findings are as follows: 1) a lesion-preparation strategy incorporating ShockFast™ IVL yielded OCT-confirmed calcium fractures in 71% of treated lesions (95% CI 45–88%), with ≥2 fractures in 43%; 2) final stent expansion exceeded 80% in the totality of cases, with no major malapposition or edge dissections recorded; and 3) final MSA was 4.77 mm^2^ (IQR 3.59–5.34). Taken together, these data provide the first serial OCT insights into calcium modification following a lesion-preparation strategy incorporating ShockFast™ IVL in severely calcified coronary disease.

The acoustic pressure wave mechanism supporting IVL-mediated calcium fracture is well characterized from OCT sub-studies of the Shockwave™ C2 system. In the DISRUPT CAD III and IV OCT sub-analyses, calcium fractures were confirmed in 79–82% of treated lesions, with multiple fractures in 52–67% of cases, establishing the structural basis for the improved vessel compliance and stent deployment observed following IVL therapy [14,15,25]. Our findings with the ShockFast™ system–a fracture rate of 71% with ≥2 fractures in 43% of lesions—fall within the range reported in published Shockwave™ OCT sub-studies, although these indirect comparisons are inevitably limited by differences in patient selection, lesion complexity, predilatation strategy, imaging protocol, OCT analysis methodology, and core-laboratory adjudication across studies. Our observations are consistent with the hypothesis that the dual-emitter design of the ShockFast™ catheter generates acoustic energy adequate for calcium fracture across a range of calcification morphologies, but do not establish equivalence or comparative efficacy. This is particularly noteworthy given that 57% of our cohort harbored calcified nodules—36% of which were eruptive—which are recognized as one of the most resistant calcium subtypes for lesion preparation and have been associated with higher rates of stent failure [26].

The fracture depth distribution observed in our cohort, with 70% of cases presenting full-thickness (transmural) fractures, is of hypothesis-generating mechanistic relevance. Transmural fractures have been associated with greater improvement in vessel compliance and more favorable stent expansion compared with superficial fractures in Shockwave™ OCT sub-studies [15]. The proportion of transmural fractures in our series is consistent with the ShockFast™ dual-emitter design being capable of generating acoustic pressure sufficient to traverse the full calcium depth, particularly in lesions with high calcium thickness, though without head-to-head comparison no claim of superiority or equivalence can be made.

The stent optimization results are favorable. Median SE of 89.5% with SE >80% in 100% of lesions is consistent with contemporary IVL benchmarks. Our results suggest that calcium fractures created by a lesion-preparation strategy incorporating ShockFast™ IVL translate into the expected improvement in vessel compliance and DES deployment quality. The complete absence of major malapposition and edge dissections further supports the safety of the ShockFast™ system from an imaging standpoint.

The exploratory correlative analyses presented in Table 7 are hypothesis-generating only and must be interpreted in light of the very small sample size, which renders all such analyses severely underpowered. Two cautions follow. First, the strongest observed association—a positive correlation between post-lesion-preparation lumen gain, measured by OCT immediately following IVL completion and prior to any additional balloon dilatation or stent implantation, and final MSA (r_s_ = 0.85, *p* = 0.0002, uncorrected and descriptive)—cannot be regarded as confirmatory. No correction for multiple comparisons was applied and no multivariable adjustment was feasible. With only 14 observations this coefficient is inherently unstable and may be driven by individual outliers or by shared dependence on vessel size, and it should not be interpreted as evidence of a causal relationship. Second, and equally important, the absence of a detectable correlation between baseline calcium morphology parameters (calcium arc, thickness, length, or OCT calcium scores) and stent optimization outcomes must not be interpreted as evidence of no true relationship. With only 14 patients, the failure to detect an association is highly likely to reflect insufficient statistical power rather than a genuine absence of a physiological relationship. This correlation may in part reflect shared dependence on vessel size and overall lesion expandability rather than a causal relationship between IVL-mediated lumen enlargement and final stent optimization. The observation is nonetheless consistent with the mechanistic framework in which IVL-induced calcium fractures and resulting improvement in vessel compliance enable subsequent stent expansion [14,15]. Confirmation in larger, adequately powered datasets with appropriate adjustment is required before this observation can inform clinical decision-making.

Final stent optimization metrics were broadly comparable between fracture-positive and fracture-negative lesions in our cohort, a finding that aligns with prior OCT sub-analyses of the Shockwave™ platform in which the presence of OCT-confirmed fractures has not been consistently associated with measurable differences in final MSA [14,15]. Although the published Shockwave™ literature has additionally reported a relationship between greater calcium arc and a higher number or depth of fractures, the present cohort was underpowered to confirm such a relationship (r_s_ = 0.06, *p* = 0.84 for calcium arc vs. fracture presence; Table 7).

Calcium eccentricity is a clinically relevant morphologic feature that may influence IVL effectiveness, with differences in fracture yield reported between concentric and eccentric calcium distributions [27,28]. In the present cohort, formal computation of an eccentricity index at the maximum calcium site would have required re-analysis of all OCT runs, and stratified inference in 14 lesions would have been statistically uninformative; this represents a recognized limitation and a priority for future, adequately powered ShockFast™ OCT studies.

Calcified nodules merit specific consideration as one of the most resistant calcium subtypes to acoustic-mediated lesion preparation. In the 8 lesions of our cohort harbouring calcified nodules, calcium fractures were either partial-thickness (non-transmural) or indeterminate (non-visible); no fully transmural fracture within the body of the calcified nodule itself was observed. This pattern is consistent with the presence of either non-visible fractures (under-resolution of OCT) or the recognised resistance of calcified nodules to acoustic-mediated lesion preparation and aligns with prior OCT observations of intravascular lithotripsy in non-eruptive calcified nodules [14].

## 5. Limitations

Several important limitations of the present sub-study warrant acknowledgment. First, the sample size, while appropriate for a mechanistic imaging sub-study of a novel device and consistent with published OCT sub-analyses of IVL systems, limits the statistical power of all exploratory analyses and precludes definitive conclusions. All quantitative findings, and particularly the exploratory correlations, should be interpreted as hypothesis-generating only; given the small sample and the number of pairwise associations examined without correction for multiple comparisons, these associations require confirmation in larger, adequately powered cohorts before any mechanistic or clinical conclusion can be drawn. Second, this is a single-center, prospective case series from a tertiary referral center, and the generalizability of results to lower-volume or less-experienced centers should be interpreted with caution. Third, OCT was acquired at all three mandatory time points in only 14 of 25 consecutive patients treated with the ShockFast™ system during the study period; exclusions were operator-dependent, and we cannot exclude residual selection bias, although the absence of OCT data in the excluded subgroup precludes a formal between-group comparison. Fourth, OCT analysis was performed offline by local investigators with independent re-review of fracture interpretations by a second institutional investigator but without independent core-laboratory adjudication; the analysts were not blinded to procedural details and outcomes, as both were involved in the index procedures. This introduces a risk of measurement bias, particularly for fracture detection and depth classification, which may have inflated the observed fracture yield. Fifth, the Revised OCT Calcium Score applied in this study was derived and validated in severely calcified lesions with a calcium arc >270°. As 9 of 14 lesions in our cohort exhibited a calcium arc below this threshold, application of the Revised Score to these lesions constitutes an extrapolation beyond its validated range, and the corresponding values should be interpreted with caution. We therefore also report the original OCT calcium score (Fujino et al.), which was validated across the full spectrum of calcium severity. Sixth, the absence of a single standardized DES platform may have introduced platform-dependent variability in malapposition measurement. Seventh, in 12 of 14 lesions predilatation occurred between Pullback 1 and Pullback 2, such that the (Pullback 2—Pullback 1) lumen change (“post-lesion-preparation lumen gain”) reflects the combined effect of predilatation and IVL rather than IVL in isolation; final stent optimization metrics similarly reflect the combined effect of the full lesion-preparation strategy of which IVL was a central component (including, in subsets of patients, rotational or orbital atherectomy, non-compliant balloon use, and post-dilatation) rather than the isolated effect of ShockFast™ IVL. Eighth, the absence of a comparator arm with the Shockwave™ IVL system precludes direct head-to-head device comparison; all comparisons with published Shockwave™ data are indirect and hypothesis-generating only. Ninth, formal sub-analyses stratified by calcium eccentricity, calcified nodule status, fracture presence, or atherectomy use were not performed given the very small cohort and would not have yielded informative inference; these represent priorities for future, adequately powered studies. Tenth, the present sub-study focuses exclusively on acute procedural and imaging outcomes; mid- and long-term clinical follow-up at 30 days and 12 months is ongoing within the Cardio-FR registry and will be reported separately.

## 6. Conclusions

In this first dedicated serial OCT sub-study of the Shunmei ShockFast™ IVL system, we observed calcium fractures in the majority of severely calcified de novo coronary lesions. The favorable stent expansion and the absence of major malapposition or edge dissections were achieved with ShockFast™ IVL as the central calcium-modification component of a comprehensive lesion-preparation strategy, rather than by IVL in isolation. These hypothesis-generating findings provide the first systematic OCT mechanistic observations on calcium modification with a lesion-preparation strategy incorporating ShockFast™ IVL, consistent with feasibility as a novel IVL platform. Larger, multicenter, comparative studies with independent core-laboratory adjudication, longer-term clinical follow-up, and direct head-to-head imaging comparison with the established Shockwave™ system are warranted to define the comparative performance of ShockFast™ in contemporary interventional practice.

## Figures and Tables

**Figure 1:**
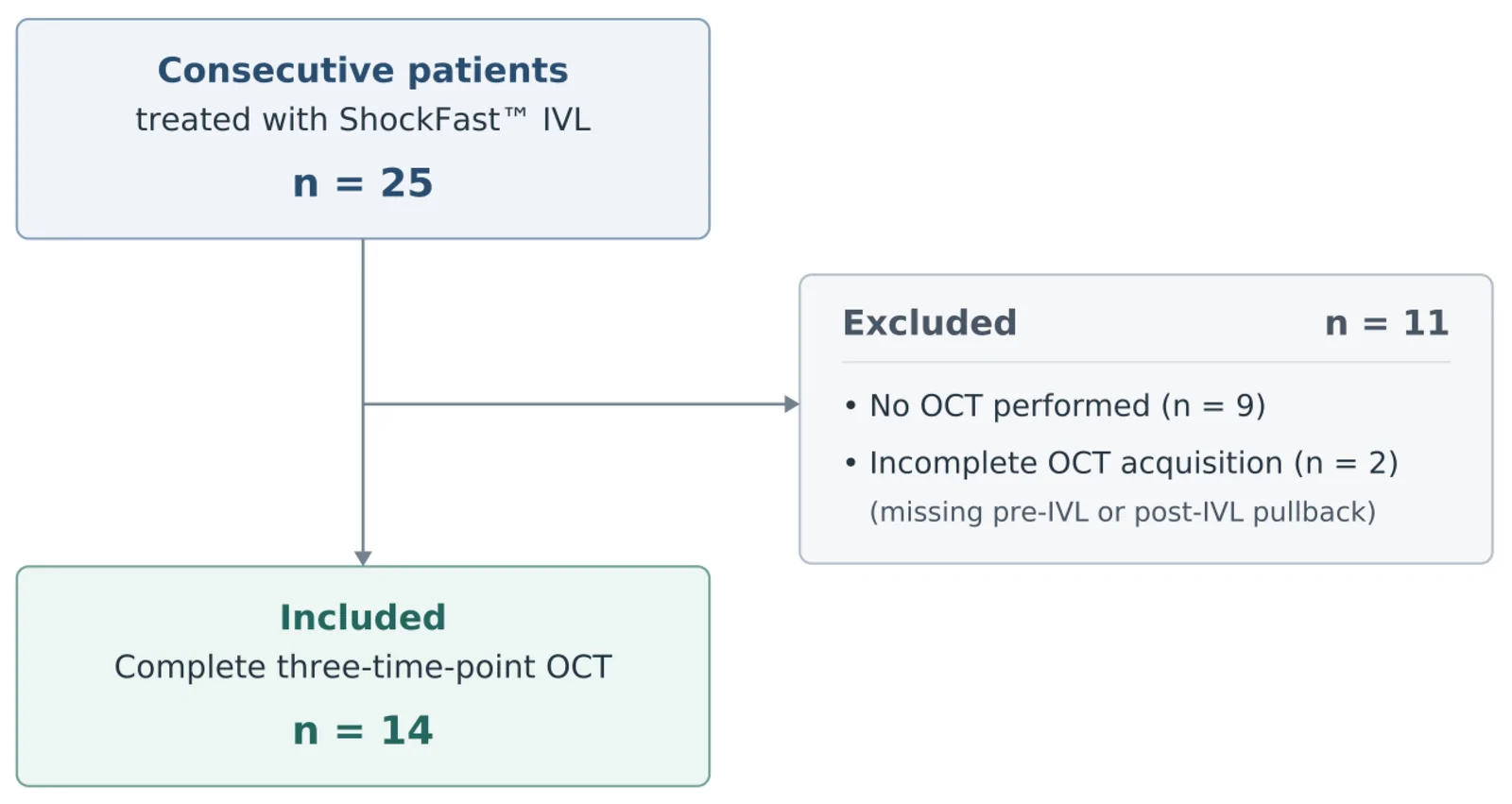
Study-flow diagram.

**Figure 2:**
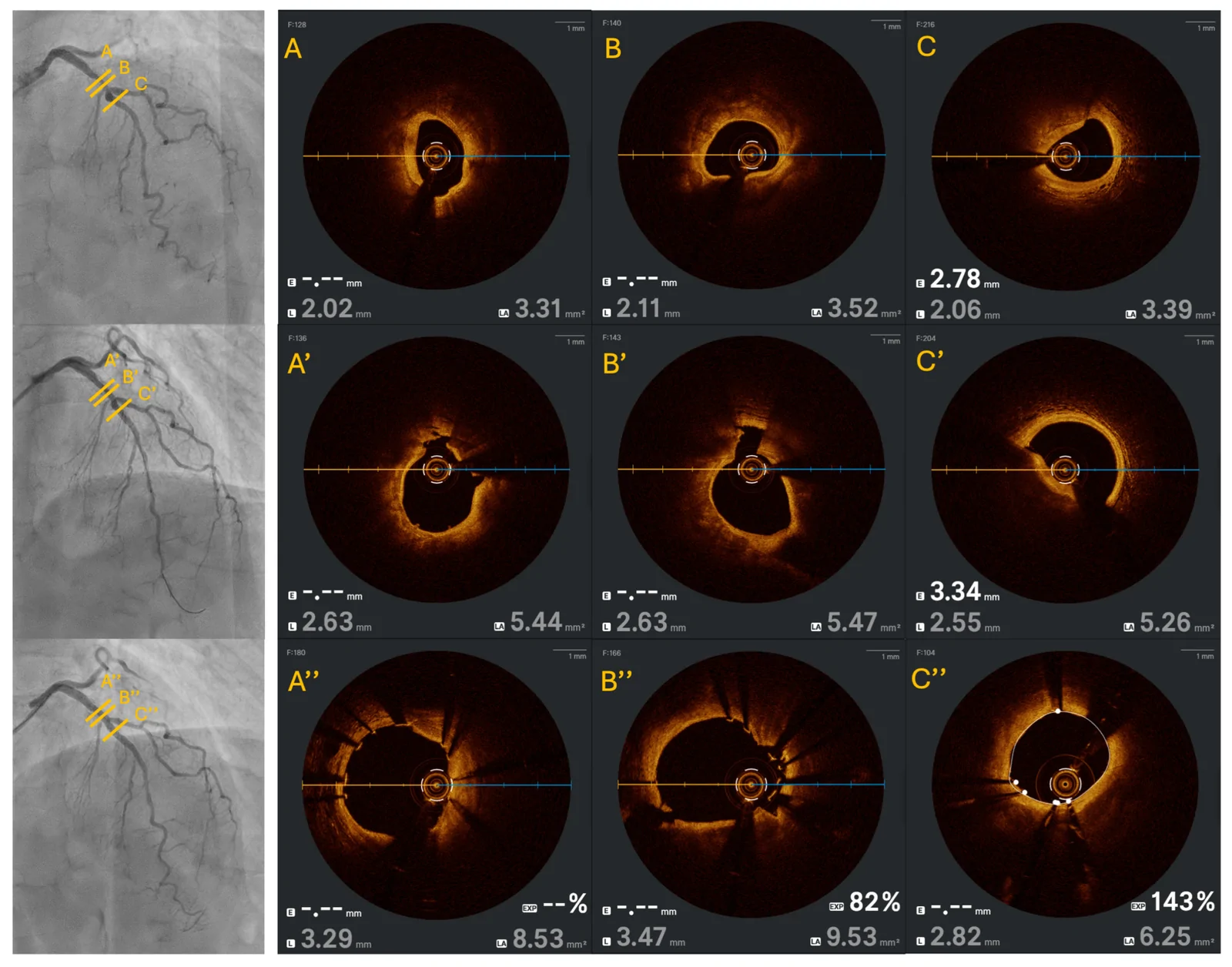
OCT case example of treatment with the Shunmei ShockFast™ IVL system. (**A**)—Baseline MLA before any device delivery; concentric mixed deep and superficial thick calcification. (**B**)—Cross-section near the MLA showing diffuse circumferential, deep and superficial, thick calcium. (**C**)—Eccentric deep calcification. (**A’**)—MLA after ShockFast™ IVL treatment with evidence of lumen gain (3.31 to 5.44 mm^2^), one full-thickness calcium fracture (12 o’clock), and one partial-thickness fracture (2–3 o’clock). (**B’**)—Same cross-section as (**B**) with a full-thickness (transmural) calcium fracture and significant lumen increase. (**C’**)—Eccentric deep calcification after ShockFast™ IVL treatment; fracture-depth assessment was inconclusive (indeterminate), with evidence of a dissection (9 o’clock) and lumen gain. (**A’’**,**B’’**)—Final OCT run after stent implantation and optimization. The stent is well apposed and expanded, with stretched calcium fractures and substantial lumen gain. (**C’’**)—Despite the absence of a clearly visualized fracture in the deep eccentric calcification, substantial lumen gain was achieved.

**Table 1:** Baseline clinical characteristics.

Variable	Value
Age, years [IQR]	74 [69–77]
Female gender, *n* (%)	3 (21)
Diabetes mellitus, *n* (%)	4 (29)
Arterial hypertension, *n* (%)	13 (93)
Dyslipidemia, *n* (%)	13 (93)
Active smoker, *n* (%)	3 (21)
Prior MI, *n* (%)	3 (21)
Prior PCI, *n* (%)	5 (36)
Prior CABG, *n* (%)	2 (14)
Family history for CAD, *n* (%)	2 (14)
**Clinical Presentation, *n* (%)**	
STEMI, *n* (%)	0 (0)
NSTEMI/UA, *n* (%)	1 (7)
CCS, *n* (%)	13 (93)
LVEF, % ± SD	57 ± 8

Data are presented as mean (SD = standard deviation), median (IQR = interquartile range) or number (*n* = number; % = percentage), as appropriate. CABG = coronary artery bypass grafting; CAD = coronary artery disease; CCS = chronic coronary syndrome; LVEF = left ventricular ejection fraction; MI = myocardial infarction; NSTEMI = non-ST-elevation myocardial infarction; PCI = percutaneous coronary intervention; STEMI = ST-elevation myocardial infarction; UA = unstable angina.

**Table 2:** Procedural characteristics.

Variable	Value
Number of vessels treated per procedure, *n* [IQR]	1 [1–1]
**Vessel treated, *n* (%)**	
RCA, *n* (%)	10 (71)
LAD, *n* (%)	4 (29)
**ACC/AHA coronary lesion calcification**	
Severe calcification, *n* (%)	14 (100)
Type B2, *n* (%)	1 (7)
Type C, *n* (%)	13 (93)
DES implantation, *n* (%)	14 (100)
Number of DES implanted, *n* [IQR]	2 [1–2]
Maximum DES diameter, mm [IQR]	3 [2.75–3.5]
DES total length, mm (±SD)	50 ± 19
DES implantation pressure, atm (±SD)	14 ± 8
DCB only strategy, *n* (%)	0 (0)
Predilatation before IVL, *n* (%)	12 (86)
SC balloon, *n* (%)	5 (38)
SC balloon max. diameter, mm [IQR]	1.5 [1.5–1.5]
SC balloon max. pressure, atm [IQR]	16 [16–18]
NC balloon, *n* (%)	11 (79)
NC balloon max. diameter, mm [IQR]	2.5 [2.0–2.5]
NC balloon max. pressure, atm [IQR]	20 [12–26]
Rotational or orbital atherectomy, *n* (%)	3 (21)
ShockFast, *n* (%)	14 (100)
ShockFast max. diameter, mm [IQR]	2.5 [2.5–3]
ShockFast max. pressure, atm [IQR]	7 [6–8]
ShockFast pulses, *n* [IQR]	120 [120–120]
Post-dilatation, *n* (%)	14 (100)

Data are mean (SD), median (IQR) or number (*n*; %), as appropriate. ACC/AHA = American College of Cardiology/American Heart Association; atm = atmosphere; DCB = drug-coated balloon; DES = drug-eluting stent; IVL = intravascular lithotripsy; LAD = left anterior descending artery; NC = non-compliant; RCA = right coronary artery; SC = semi-compliant.

**Table 3:** QCA analysis.

Variable	Value
Initial MLD, mm [IQR]	0.74 [0.54–1.13]
Distal reference diameter (RVD), mm [IQR]	2.43 [2.21–2.5]
Initial diameter stenosis, % [IQR]	86.5 [80–94]
Lesion length, mm [IQR]	32.4 [28–46.3]
Post-ShockFast MLD, mm [IQR]	1.58 [1.21–2]
Post-ShockFast residual diameter stenosis, % [IQR]	63.5 [53–73]
Final MLD, mm [IQR]	2.8 [2.3–3.3]
Acute lumen gain, mm [IQR]	1.93 [1.73–2.2]
Final diameter stenosis, % [IQR]	12 [5–23]

Data are mean (SD), median (IQR) or number (*n*; %), as appropriate. MLD = minimal lumen diameter; QCA = quantitative coronary analysis; RVD = reference vessel diameter.

**Table 4:** Baseline OCT quantitative calcium characterization (Pre-IVL).

OCT Variable	Value
**Lumen Geometry**	
Minimum lumen area (MLA), mm^2^ [IQR]	2.22 [1.4–3.61]
Minimum lumen diameter (MLD), mm [IQR]	1.58 [1.26–1.96]
Proximal reference lumen diameter, mm [IQR]	3.47 [3.04–3.98]
Distal reference lumen diameter, mm [IQR]	2.39 [2.18–2.62]
Proximal EEL mean diameter, mm [IQR]	4.16 [3.92–4.56]
Distal EEL mean diameter, mm [IQR]	3.08 [2.55–3.37]
Lesion length, mm [IQR]	44.74 [31.4–59.8]
**Calcium Morphology**	
Calcium arc, ° [IQR]	272 [200–308]
Calcium thickness, mm [IQR]	1.6 [1.12–1.83]
Calcium length, mm [IQR]	26.2 [23–43.4]
Original OCT calcium score, *n* [IQR]	4 [4–4]
Revised OCT calcium score, *n* [IQR]	3 [1–3]
**Qualitative Calcium Features**	
Calcified nodule, *n* (%)	8 (57)
Eruptive calcified nodule, *n* (%)	5 (36)

Data are mean (SD), median (IQR) or number (*n*; %), as appropriate. EEL = external elastic lamina; MLA = minimal lumen area; MLD = minimal lumen diameter; OCT = optical coherence tomography.

**Table 5:** Post-IVL OCT calcium fracture assessment.

OCT Variable	Value
**Calcium Fracture Outcomes**	
Calcium fracture(s) present, *n* (%)	10 (71, 95% CI 45–88%)
≥2 fractures, *n* (%)	6 (43)
**Fracture Depth Classification (*n* = 10 fracture-positive lesions)**	
Partial-thickness (non-transmural) fracture, *n* (%)	3 (30)
Full-thickness (transmural) fracture, *n* (%)	7 (70)
**Post-Lesion-Preparation Lumen Gain**	
Post-lesion-preparation lumen gain, mm^2^ [IQR]	0.53 [0.3–0.84]

Data are mean (SD), median (IQR) or number (*n*; %), as appropriate. Post-lesion-preparation lumen gain = (Pullback 2 MLA–Pullback 1 MLA), reflecting the combined effect of predilatation (when performed) and IVL between Pullbacks 1 and 2. Header and row relabeled from “IVL-mediated lumen gain”/“Lumen gain after IVL” to align with the agreed terminology change. IVL = intravascular lithotripsy; OCT = optical coherence tomography.

**Table 6:** Post-stenting OCT stent optimization metrics.

OCT Variable	Value
**Stent Expansion**	
Minimum stent area (MSA), mm^2^ [IQR]	4.77 [3.59–5.34]
Stent expansion (SE), % [IQR]	89.5 [85–94]
Stent expansion >80%, *n* (%)	14 (100; 95% CI 78–100%)
Acute lumen gain, mm^2^ [IQR]	2.52 [1.49–3.57]
**Stent Apposition**	
Major malapposition (>400 µm, >1 mm), *n* (%)	0 (0)
Minor malapposition, *n* (%)	6 (43)
Medial edge dissection, *n* (%)	0 (0)

Data are mean (SD), median (IQR) or number (*n*; %), as appropriate. MSA = minimum stent area; OCT = optical coherence tomography; SE = stent expansion, defined as MSA/average of proximal and distal reference lumen area × 100.

**Table 7:** Exploratory correlative analyses: Baseline OCT calcium morphology vs. post-IVL outcomes.

Correlation Pair	Spearman r_s_	*p*-Value
Calcium arc (°)—Fractures, *n* (%)	0.0589	0.8415
Calcium arc (°)—Final SE (%)	−0.0850	0.7727
Calcium arc (°)—Final MSA (mm^2^)	−0.1980	0.4974
Calcium thickness (mm)—Fractures, *n* (%)	0.1376	0.6390
Calcium thickness (mm)—Final SE (%)	0.2199	0.4500
Calcium thickness (mm)—Final MSA (mm^2^)	0.5132	0.0605
Revised OCT calcium score—Final SE (%)	−0.2305	0.4278
Revised OCT calcium score—Final MSA (mm^2^)	0.0642	0.8273
Post-lesion-preparation lumen gain (mm^2^)—Final MSA (mm^2^)	0.8516	0.0002
Original OCT calcium score—Final SE (%)	−0.3033	0.2918
Original OCT calcium score—Final MSA (mm^2^)	0.2375	0.4136
Calcium length (mm)—Fractures, *n* (%)	−0.3138	0.2746
Calcium length (mm)—Final MSA (mm^2^)	−0.3143	0.2738

IVL = intravascular lithotripsy; MSA = minimum stent area; OCT = optical coherence tomography; SE = stent expansion, defined as MSA/average of proximal and distal reference lumen area × 100.

## Data Availability

The raw data supporting the conclusions of this article will be made available by the authors on request.

## Abbreviations

ACC/AHA	American College of Cardiology/American Heart Association
BMI	body mass index
CABG	coronary artery bypass grafting
CAD	coronary artery disease
CCS	chronic coronary syndrome
CK	creatine kinase
CK-MB	creatine kinase MB
DCB	drug-coated balloon
DES	drug-eluting stent
ECG	electrocardiogram
EEL	external elastic lamina
IQR	interquartile range
IVL	intravascular lithotripsy
LAD	left anterior descending artery
LVEF	left ventricular ejection fraction
MACE	major adverse cardiovascular events
MI	myocardial infarction
MLA	minimal lumen area
MLD	minimal lumen diameter
MSA	minimal stent area
NC	non-compliant
NSTEMI	non-ST-elevation myocardial infarction
OCT	optical coherence tomography
PCI	percutaneous coronary intervention
QCA	quantitative coronary analysis
RCA	right coronary artery
RVD	reference vessel diameter
SC	semi-compliant
STEMI	ST-elevation myocardial infarction
UA	unstable angina
